# Cruising with an epigenetic brake: role of DNA methylation in maintaining immune homeostasis in Soybean

**DOI:** 10.1093/plcell/koag078

**Published:** 2026-03-16

**Authors:** Meenu Singla-Rastogi, Pei Qin Ng

**Affiliations:** Assistant Features Editor, The Plant Cell, American Society of Plant Biologists; Department of Biology, Indiana University, Bloomington, IN 47405, United States; Assistant Features Editor, The Plant Cell, American Society of Plant Biologists; Department of Plant Sciences, University of Cambridge, Cambridge CB2 3EA, United Kingdom

Plants constantly walk a fine line between growing and defending themselves. Strong immune responses are essential for survival, but they come at a cost. When defense pathways are switched on too early or for too long, it results in severe penalties to plant growth and overall fitness (Deleris et al. 2016). Epigenetic regulation mechanisms, such as DNA methylation, have long been thought to help manage this growth–defense tradeoff, yet clear links between DNA methylation and immune gene control have been surprisingly hard to pin down. This is especially true in *Arabidopsis thaliana*, as changes in DNA methylation often prompt limited effects on gene expression owing to the relatively low transposable element (TE) density in its genome (Dowen et al. 2012). In plants, RNA-directed DNA methylation (RdDM) is a key epigenetic regulatory pathway that typically relies on RNA Polymerase (Pol) IV-derived 24-nt small RNA (sRNA) to direct DNA methylation toward regions such as TEs, while RdDM is also achievable using Pol V-derived siRNAs via transcription of noncoding RNAs (Gallego-Bartolomé 2020), likely indirectly. In *Glycine max* (soybean), the function of these 2 plant-specific polymerases in driving RdDM has yet to be explored together in an RNA-silencing context.

Here we present 2 independent studies recently published in *The Plant Cell* investigating Pol IV- and Pol V-derived sRNA-mediated RdDM in soybean, a crop with a large, transposon-rich genome composed of more than 50% TEs (Schmutz et al. 2010; Wang et al. 2023). In their new work, **Zhang and colleagues (2026)** used CRISPR/Cas9 genome editing to generate knockout mutants of GmNRPD1 (Pol IV largest subunit) and GmNRPE1 (Pol V largest subunit) to uncover a central role for RdDM in balancing soybean immune responses. In a separate study, **Wang and colleagues (2026)** generated RNAi lines targeting GmNRPD1, GmNRPE1, and the shared second-largest subunit GmNRP(D/E)2. Together, these complementary genetic approaches reveal a central role for RdDM in maintaining immune homeostasis in soybean.

In both studies, significant developmental phenotypes were noted in respective lines, including pronounced developmental phenotypes upon disruption of Pol IV or Pol V. The CRISPR null mutants of *GmNRPD1* (*gmnrpd1-1*, *gmnrpd1-2*) and *GmNRPE1* (*gmnrpe1-1*, *gmnrpe1-2*) displayed severe dwarfism and growth retardation. Similarly, RNAi lines targeting *GmNRPE1* or *GmNRP(D/E)2* exhibited delayed growth under long-day conditions, and *GmNRPE1*-RNAi plants showed reduced seed set and smaller seeds, while notably GmNRPD1 RNAi lines did not show developmental phenotypes. These phenotypes underscore the importance of Pol IV and Pol V in soybean growth and development.

Next, to assess how Pol IV and Pol V affect sRNA production, Zhang et al. (2026) performed sRNA-seq using the null mutants of *GmNRPD1* and *GmNRPE1*. As expected, loss of GmNRPD1 caused a dramatic depletion of 24-nt siRNAs, while *GmNRPE1* mutants showed a milder decrease, consistent with their respective roles in the RdDM pathway (Matzke and Mosher 2014). Interestingly, both mutants also accumulated 21- and 22-nt sRNAs, suggesting compensatory or noncanonical sRNA responses at RdDM target loci (Fig. 1a).

**Figure 1:**
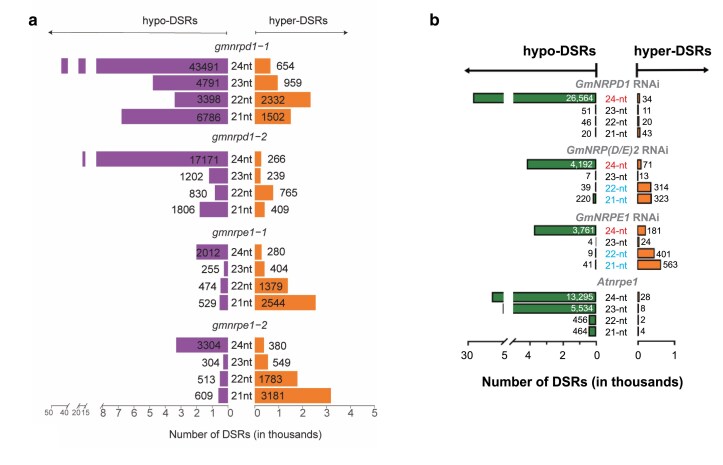
Effects of pol IV and V knockout and knockdown on sRNA accumulation in soybean mutants. **a)** Identification of hyper- and hypo-DSRs for each size class (21 to 24 nt) in CRISPR mutants of *GmNRPD1* and *GmNRPE1* relative to wild type (WT). The genome was divided into non-overlapping 100-base windows, and rRNA-normalized sRNA abundance within each window was compared between mutants and WT. A fold change (FC) ≥ 1.5, and a false discovery rate (FDR) < 0.05 was used as thresholds. Panel from Fig. 2C of Zhang et al. 2026. **b)** Identification of DSRs for each size class between transgenic RNAi (T) and nontransgenic (NT) lines for *GmNRPD1*, *GmNRP(D/E)2*, and *GmNRPE1*. The genome was tiled into 100-bp non-overlapping windows, and rRNA-normalized sRNA abundance was compared between the T and NT samples for each window. A fold change (FC) ≥ 2 and adjusted *P*-value <0.01 was used as thresholds. Panel from Fig. 2A of Wang et al., 2026.

Consistently, Wang et al. (2026) reported similar observations with sRNA-seq analysis of their RNAi lines. While 24-nt siRNA levels were globally reduced in *GmNRPD1* and *GmNRP(D/E)2* RNAi lines, the *GmNRPE1* RNAi lines were not affected (Fig. 1b). After further categorization of hypo-differential small RNA regions (DSRs) into Pol IV-dependent, Pol V-dependent, and Pol IV-V shared subsets, the authors gained clearer insights into the sRNA accumulation at different DSRs. The authors also reported enriched 21- and 22-nt sRNAs in *GmNRP(D/E)2* and Gm*NRPE1* but not in *GmNRPD1* RNAi lines. This was not observed in *nrpe1* mutants of other plants such as *Arabidopsis*, *Brasica rapa*, and *Solanum lycopersicum*, suggesting this phenomenon is unique to soybean Pol V (Wang et al. 2026). These 21- to 22-nt Gm*NRPE1* dependent hyper-DSRs corresponded to TE (213 DSRs) and coding regions (66 DSRs). Genes associated with the 21- to 22-nt Gm*NRPE1* dependent hyper-DSRs were over-represented in Gene Ontologies (GOs) for functions in “defense response” and “Leucine-rich-repeats.”

In Zhang et al. (2026), the impact of RdDM disruption on the methylome was examined using whole-genome bisulfite sequencing. This analysis revealed widespread loss of non-CG DNA methylation, particularly CHH methylation, at thousands of RdDM-controlled regions in null mutants of *GmNRPD1* and *GmNRPE1*. A substantial subset of the differentially methylated regions was enriched in TEs—especially LTR retrotransposons—located close to genes. Similarly, Wang et al. (2026) also reported a significant reduction of non-CG DNA methylation in Gm*NRPD1* RNAi lines, with significant reduction at CG, CHG, and CHH sites in *GmNRP(D/E)2* and Gm*NRPE1* RNAi lines at 24-siRNA hypo-DSRs. Taken together, both studies suggest that soybean Pol V is key in DNA methylation of CG, CHG and CHH.

In both studies, the physiological relevance of this epigenetic misregulation in the mutants was tested using pathogen infection assays. Both *GmNRPD1* and *GmNRPE1* null mutants exhibited severe dwarfism and growth retardation, reminiscent of autoimmune phenotypes. Unsurprisingly, both mutants displayed enhanced resistance to the oomycete pathogen *Phytophthora sojae*. Thus, RdDM-mediated repression of immune genes is not merely a passive silencing mechanism but also a crucial safeguard that allows plants to grow normally while retaining the capacity for rapid defense activation. Importantly, this study revealed that RdDM is dynamically modulated during pathogen infection. In wild-type soybean, *P. sojae* challenge led to transcriptional downregulation of core RdDM components, accompanied by loss of CHH methylation at RdDM-targeted defense genes to promote their robust induction. This suggests that soybean primarily employs a passive release of RdDM-mediated repression, rather than active DNA demethylation, to reprogram immune gene expression during infection (Zhang et al. 2026). Wang et al. (2026) similarly observed significant reductions in *P. sojae* colonization across all RNAi lines, with *GmNRP(D/E)2*-RNAi showing the strongest effect.

Overall, both studies establish RdDM as a key regulator of immune homeostasis in soybean and illustrate how genome architecture amplifies the biological impact of epigenetic regulation. They also collectively demonstrate that RdDM acts as an “epigenetic brake,” which prevents immune signaling pathways from accelerating when they are not needed. These findings open the door to future strategies to manipulate RdDM to enhance disease resistance in crops while minimizing the growth penalties typically associated with constitutive immune activation.

## Recent related articles in the *The Plant Cell*:

- Vaucheret and Voinnet (2024) reviewed the current understanding of the biogenesis of diverse sRNAs in plants and their relative functions in different stages of plant growth, including 24-nt and 21- to 22-nt sRNAs featured in this In Brief.
- Dodds et al. 2024 reviewed the key principles and players of plant immunity, including LRRs, and highlighted the research that leads to these insights.
- Zhang et al. 2025 investigated the molecular basis of CG maintenance methylation by plant METHYLTRANSFERASE 1 (MET1), illuminating the molecular basis of MET1 autoinhibition and its preference for hemimethylated DNA substrates.

## Data Availability

No new data were generated or analyzed in support of this research.

## Dive Curated Terms

The following phenotypic, genotypic, and functional terms are of significance to the work described in this paper:

MET1 Gramene: AT5G49160
MET1 Araport: AT5G49160

NRPE1 Gramene: AT2G40030
NRPE1 Araport: AT2G40030
